# Elimination of diarrheal mortality in children – the last half million

**DOI:** 10.7189/jogh.09.020102

**Published:** 2019-12

**Authors:** Mathuram Santosham, Christopher P Duggan, Roger Glass

**Affiliations:** 1 Departments of International Health and Pediatrics, Johns Hopkins University, Baltimore, Maryland, USA; 2 International Vaccine Access Center; Johns Hopkins University, Baltimore, Maryland, USA; 3 Center for American Indian Health, Johns Hopkins University, Baltimore, Maryland, USA; 4 Departments of Pediatrics, Harvard Medical School, Boston, Massachusetts, USA; 5 Nutrition and Global Health and Population, Harvard TH Chan School of Public Health, Boston, Massachusetts, USA; 6 Director, Fogarty International Center, National Institutes of Health, Bethesda, Maryland, USA

This issue of the journal includes two papers discussing the extraordinary 90% decline in childhood deaths from diarrhea over the past four decades. Black and colleagues use an analytic tool to partition the contribution of the many advances made in diarrheal disease control, and outline programs and policies that have led to this public health success [1]. Wolfheim and colleagues recount the history of World Health Organization (WHO)’s Control of Diarrheal Diseases (CDD) program, which played a major role in this achievement [2]. Indeed, the number of deaths from diarrhea among children under age 5 years has dropped from more than 5 million per year to approximately 500 000, despite a growing population of children globally.

The cornerstone of the CDD program, and the breakthrough in clinical treatment, was the discovery of Oral Rehydration Therapy (ORT) and its introduction into child health programs worldwide. The original stimulus for the development of ORT was the recognition that while cases of severe dehydrating cholera could be treated with IV therapy in hospitals and clinics, many patients with diarrhea did not have access to health facilities, and many health facilities lacked the necessary supplies or trained personnel to deliver IV fluids. ORT was initially evaluated in cholera patients in hospitals and proven to be effective [3]. Subsequently ORT was tested and shown to be effective in diarrhea of all etiologies and in all ages. Other important components of the CDD program included the promotion of feeding during diarrhea (including continued breastfeeding for at least 6 months of life), and a robust research agenda supporting studies documenting the value of zinc in diarrhea treatment, early work on vaccines for the most severe common agents of diarrhea (rotavirus, typhoid, cholera, and shigella), and an emphasis on adequate nutrition.

In 1978, on the 10th anniversary of the discovery of ORT, and after 10 years of research on different formulations, strategies of program implementation, and massive training of health workers, the *Lancet* heralded the physiologic basis of ORT as “potentially the most important medical advance of this century” [4]. It was also a prime example of “reverse transfer of technology” [5] where novel treatments first tested and evaluated in developing countries were subsequently adopted and widely used in the United States (US) and other western countries. Even in the US, ORT studies were first conducted on the White Mountain Apache Indian reservation [6,7] and went on to be recommended by the American Academy of Pediatrics and US Centers for Disease Control and Prevention for the routine treatment of diarrhea in American children [8].

Other lessons were learned along the way. In the 1970s, standard practice for the treatment of diarrhea was to “rest the gut” and withhold regular feeding for several days after the onset of symptoms. This practice was found to aggravate the diarrhea-malnutrition-infection cycle. In the mid 1980s randomized trials of early feeding on the Apache Indian reservation [9] and subsequently in several countries [10,11] demonstrated that continued feeding during diarrhea should be accepted as first line of treatment in all countries, including high income countries [8,12,13].

In the 1970s and 1980s, more than 20 new pathogens were discovered to be causal agents of childhood diarrhea and rotavirus was soon crowned as the most common and severe pathogen. Studies of rotavirus documented solid natural immunity that developed following infections early in life, observations that laid down the challenge to develop vaccines that could imitate this immunity. Unlike many of the bacteria and parasites that were found in disease outbreaks and in unsanitary environments, rotavirus infected all children worldwide, so vaccines provided the only feasible strategy for prevention. Vaccine development followed a rocky road but in 2006, two live oral vaccines completed phase 3 clinical trials and were licensed for use. WHO recommended Rotateq (Merck) for global use in 2006 in countries with substantial childhood deaths from diarrhea and Rotarix (GSK) was added to the recommendation in 2009. GAVI came forward to purchase these vaccines for use in low-income countries (LICs), a decision that rapidly facilitated their uptake. By 2018, these vaccines had been introduced into the national immunization programs of more than 90 countries. Nevertheless, more than 50% of the world’s children still do not have access to rotavirus vaccine.

The impact to reduce hospitalizations for severe diarrhea was evident within 2 years of vaccine introduction and in several countries (eg, Mexico, Brazil, Bolivia, Honduras, Panama, Venezuela and Malawi), deaths from diarrhea decreased substantially following vaccine introduction [14]. Several new live oral vaccines have been introduced recently and others are in development. Therefore, there should be an adequate global supply soon. The global impact noted to date on mortality has been relatively small since introductions are still ongoing and vaccine coverage has been less than the levels reached for other childhood vaccines. However, surveillance of hospitalization in more than 60 countries has documented that rotavirus was responsible for an average of 36% of admissions before vaccine introduction suggesting that this vaccine could have a huge impact both on hospitalizations as well as the prevention of deaths. Newly licensed vaccines for cholera and typhoid are just becoming more widely available and could in the future further decrease the burden of these enteric pathogens.

In the 1990s, the CDD program merged with other WHO programs and eventually was blended into the Maternal, Newborn, Child and Adolescent Health Research and Development Team, mirroring the amalgamation of diarrhea management into the Integrated Management of Childhood Illness (IMCI) guidelines. While this integration was of course beneficial on many fronts, the program lost focus. In the 1980s, WHO’s CDD Program had 24 dedicated staff (12 of them technical) but with IMCI, no staff member has been specifically assigned to monitor and evaluate the diarrhea control program.

While the achievement of reducing childhood death due to diarrhea is commendable, the global community cannot rest on its laurels. Several observations point need to be considered to achieve greater control:

1. Despite the great decline in childhood deaths from diarrhea, the burden of childhood diarrhea deaths remains greatest in low-income countries of South Asia and Sub-Saharan Africa. This burden of deaths falls disproportionately on poorer communities within these countries, as well as among girls more than boys [15].
2. Diarrhea illnesses are associated with other consequences including growth faltering that itself contributes to even more mortality and morbidity [16], suboptimal neurodevelopment of the child [17] and health sequelae into adulthood [18].
3. The ability to devise programs for the prevention and treatment of childhood diarrhea have relied upon the sustained and mutually respectful relationships between high- and low- and middle-income partners and scientists involved in collaborative research and clinical trials. Continuous support from the WHO, UNICEF, GAVI, many development agencies and NGOs have helped implement the scientific discoveries of ORT, zinc supplementation and novel vaccines. The impact of training programs, infrastructure support and local political engagement with country leaders and health administrators cannot be overestimated, as part of this success story, as well as the key role of multinational and government agencies.

Research and practice over the past 40 years have led to the introduction of rotavirus vaccine to reduce the incidence of severe childhood diarrhea, and treatment with ORT and zinc supplements. When used properly (and together with intravenous fluids for severe cases), these interventions should be able to prevent most diarrheal deaths. Other strategies for prevention and treatment of diarrhea are widely available and their aggressive implementation in the Global Action Plan for Pneumonia and Diarrhea (GAPPD) will be critical to meet the Sustainable Development Goal of reducing under 5 mortality to less than 25 per 1000 live births. International funders need to ensure that adequate funding is provided to WHO and country programs to hire personnel, implement and monitor the GAPPD strategy.

Today, most diarrheal deaths can be prevented with rapid treatment. We hope that the lessons learned from the decades long fight against childhood diarrhea will be reignited to eliminate the last half million deaths. Failure to do so will result in the additional loss of another 5 million children due to diarrhea in the next decade.

## References

[1] Black RE Fontaine O Lamberti L Bhan M Huicho L El Arifeen S Control of childhood diarrhea mortality 1980-2015 and interventions to eliminate preventable diarrhea deaths by 2030. J Glob Health. 2019;9:020801 10.7189/jogh.09.020801 PMC6815873 31673345

[2] Wolfheim C Fontaine O Merson M Evolution of the World Health Organization’s programmatic actions to control diarrheal diseases. J Glob Health. 2019;9:020802 . 10.7189/jogh.09.020802 PMC6816052 31673346

[3] Nalin DR Cash RA Islam R Molla M Phillips RA Oral maintenance therapy for cholera in adults. Lancet. 1968;292:370-3. 10.1016/S0140-6736(68)90591-6 4173788

[4] Water with sugar and salt. Lancet. 1978;2:300-1. 79090

[5] Santosham M Oral rehydration therapy: reverse transfer of technology. Arch Pediatr Adolesc Med. 2002;156:1177-9. 10.1001/archpedi.156.12.1177 12444824

[6] Hirschhorn N The treatment of acute diarrhea in children. An historical and physiological perspective. Am J Clin Nutr. 1980;33:637-63. 10.1093/ajcn/33.3.637 6766662

[7] Santosham M Burns B Nadkarni V Foster S Garrett S Croll L Oral rehydration therapy for acute diarrhea in ambulatory children in the United States: a double-blind comparison of four different solutions. Pediatrics. 1985;76:159-66. 4022687

[8] Duggan C Santosham M Glass RI The management of acute diarrhea in children: oral rehydration, maintenance, and nutritional therapy. MMWR Recomm Rep. 1992;41:1-20. 1435668

[9] Santosham M Foster S Reid R Role of soy-based, lactose-free formula during treatment of acute diarrhea. Pediatrics. 1985;76:292-8. 4022702

[10] Brown KH Dietary management of acute childhood diarrhea: optimal timing of feeding and appropriate use of milks and mixed diets. J Pediatr. 1991;118:S92-8. 10.1016/S0022-3476(05)81434-9 2007962

[11] Grange AO Santosham M Ayodele A Lesi F Stallings R Brown K Evaluation of a maize-cowpea-palm oil diet for the dietary management of Nigerian children with acute, watery diarrhea. Acta Paediatr. 1994;83:825-32. 10.1111/j.1651-2227.1994.tb13153.x 7981559

[12] Duggan C Nurko S “Feeding the gut”: the scientific basis for continued enteral nutrition during acute diarrhea. J Pediatr. 1997;131:801-8. 10.1016/S0022-3476(97)70024-6 9427881

[13] King CK Glass R Bresee JS Duggan C Centers for Disease C, Prevention. Managing acute gastroenteritis among children: oral rehydration, maintenance, and nutritional therapy. MMWR Recomm Rep. 2003;52:1-16. 14627948

[14] ROTA Council. Rotavirus: common, severe, devastating, preventable. 2016.

[15] Million Death Study Collaborators Bassani DG Kumar R Awasthi S Morris SK Paul VK Causes of neonatal and child mortality in India: a nationally representative mortality survey. Lancet. 2010;376:1853-60. 10.1016/S0140-6736(10)61461-4 21075444 PMC3042727

[16] Troeger C Colombara DV Rao PC Khalil IA Brown A Brewer TG Global disability-adjusted life-year estimates of long-term health burden and undernutrition attributable to diarrhoeal diseases in children younger than 5 years. Lancet Glob Health. 2018;6:e255-69. 10.1016/S2214-109X(18)30045-7 29433665 PMC5861379

[17] Sudfeld CR McCoy DC Danaei G Fink G Ezzati M Andrews KG Linear growth and child development in low- and middle-income countries: a meta-analysis. Pediatrics. 2015;135:e1266-75. 10.1542/peds.2014-3111 25847806

[18] DeBoer MD Chen D Burt DR Ramirez-Zea M Guerrant RL Stein AD Early childhood diarrhea and cardiometabolic risk factors in adulthood: the Institute of Nutrition of Central America and Panama Nutritional Supplementation Longitudinal Study. Ann Epidemiol. 2013;23:314-20. 10.1016/j.annepidem.2013.03.012 23608305 PMC4431615

